# Increase in Imported Dengue, Germany, 2001–2002

**DOI:** 10.3201/eid1005.030495

**Published:** 2004-05

**Authors:** Christina Frank, Irene Schöneberg, Gérard Krause, Hermann Claus, Andrea Ammon, Klaus Stark

**Affiliations:** * Robert Koch Institute, Berlin, Germany

**Keywords:** Dengue fever, Epidemiology, Surveillance, Thailand, Brazil, Travel medicine

## Abstract

Dengue fever is a reportable disease in Germany. Surveillance data from 2001 and 2002 were analyzed and compared to travel patterns. Imported dengue fever increased strongly in this time. Most infections were acquired in Southeast Asia, specifically Thailand. The 2002 epidemic in Brazil was also reflected in these data.

Dengue fever is endemic in many tropical regions worldwide (*1*,*2*). The disease is caused by any of four serotypes of dengue virus, a flavivirus. The World Health Organization estimates that 50 million infections and 22,000 dengue-related deaths occur annually. In many countries, the incidence of dengue fever in 2002 increased compared to previous years. The geographic range of dengue continues to expand, as a current large outbreak in Australia’s Northern Queensland demonstrates (*3*).

Travel to dengue-endemic areas carries the risk of acquiring the disease. Each year, an estimated 3 million German residents spend time in such countries. Country-specific risk for travel-associated dengue fever needs to be monitored to focus pretravel advice. In the absence of data on the true incidence in travelers (including asymptomatic infections and those not coming to medical attention in Germany), cases of symptomatic imported dengue fever diagnosed in Germany indicate temporal and geographic trends in all travel-associated dengue infections.

An improved surveillance system for mandatory case reporting of infectious diseases, including dengue, was implemented in Germany in January 2001. Under the Infectious Disease Control Act, German laboratories must notify local public health authorities of test results fulfilling the case definition for acute dengue virus infection, i.e., detection of viral antigen or RNA, a fourfold or greater increase in antibody titers between acute- and convalescent-phase serum samples, or detection of immunoglobulin (Ig) M antibodies to a dengue virus. The inclusion of positive IgM test results, typically the first laboratory evidence to indicate infection, enhances timeliness of reporting. In most cases, paired serum samples are tested. Local authorities then gather additional information on the patient (clinical signs, demographics, travel destination). If the case definition is fulfilled (clinical dengue plus definitive or probable laboratory evidence), the case is reported through state authorities to the central database at the Robert Koch Institute.

## The Study

We analyzed surveillance data on temporal trends, demographics, and country of infection from January 2001 to December 2002. Using recent air travel data, we calculated relative country-specific dengue fever risks for travelers from Germany. Information on the numbers of air travelers from Germany to foreign destinations in 2002 is available from the Federal Statistical Office (Statistisches Bundesamt, air tourism statistics, 2002), which receives reports on ticketed destinations from all airlines that board passengers at German airports. The Thailand Authority of Tourism provides monthly statistics on the number of visitors arriving from Germany in 2002 (available from: http://www.tat.or.th/stat/download.htm), as does the Brazilian Tourist Office for 2001 (available from: http://www.brazil.org.uk/page.php?cid=1195). On the basis of likely month of infection, monthly risk for dengue per 100,000 travelers from Germany to Thailand and Brazil was calculated.

**Figure 1:**
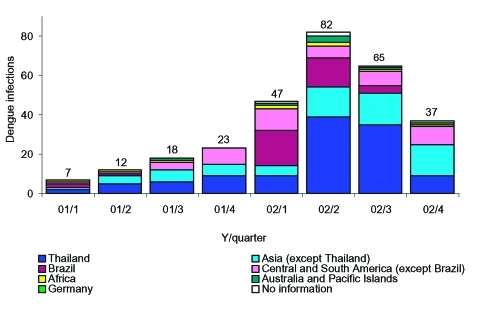
Cases of dengue fever reported in Germany 2001 (n = 60) and 2002 (n = 231) by region of acquisition.

Sixty cases meeting the case definition were reported in 2001, and 231 cases were reported in 2002. Case reports rose continuously from 7 cases in the first quarter of 2001 to 82 cases in the second quarter of 2002 (Figure 1). In both years, 55% of cases were male. The median age at infection was 37.5 years (range 20–62 years) in 2001 and 34 years (range 5–71 years) in 2002. Travelers to Thailand (n = 114, median age 31.5 years) were significantly younger than travelers to Brazil (n = 40, median age 40 years) (p < 0.001). Six cases had hemorrhagic signs, but none fulfilled the World Health Organization case definition for dengue hemorrhagic fever or dengue shock syndrome. No deaths from dengue fever were reported.

Information on travel history was available for all cases in 2001 and for 98% of 2002 cases. Overall, 39.4% of infections were acquired in Thailand (2001: 36.1%, 2002: 40.6%). In 2001, stays in Venezuela (8.3%), India (6.6%), and Cambodia (6.6%)—all countries endemic for dengue fever—were also frequently implicated. The proportion of cases imported from Brazil rose from 4.9% in 2001 to 15.5% in 2002 (p = 0.05). In the first half of 2002, 25.6% of all cases were associated with travel to Brazil. Cases imported from Thailand peaked in the second and third quarter of 2002. Venezuela contributed 1.7% of cases in 2002, compared to 8.3% in 2001 (p = 0.02). A case of nosocomial dengue virus transmitted by needlestick injury was observed in a German hospital nurse in 2002 (*4*). Relatively high country- or area-specific incidence rates among German travelers were noted for Thailand (27.9/100,000 travelers), Brazil (22.8/100,000), South America except Brazil (21.1/100,000), the Lesser Antilles (19.1/100,000), the Central American mainland south of Mexico (16.3/100,000), the former Indochina (15.2/100,000), and Indonesia (14.8/100,000) (Table).

**Table:** Regional risk for dengue fever in German travelers, 2002

Region	Country or area	No. of travelers flying in from Germany	Mentions of destination(s) among dengue patients	Incidence (cases/100,000 air travelers from Germany)
Southeast Asia	Thailand	347,569	97	27.9
South America	Brazil	162,264	37	22.8
South America	Colombia, Venezuela, Suriname, Trinidad and Tobago, Aruba, Curaçao, Bonair	61,739	13	21.1
Central America	Lesser Antilles Islands^a^	20,989	4	19.1
Central America	Guatemala, Honduras, Belize, El Salvador, Nicaragua, Costa Rica, Panama	24,564	4	16.3
Southeast Asia	Laos, Vietnam, Cambodia (Indochina)	65,963	10	15.2
Southeast Asia	Indonesia	88,053	13	14.8
Africa	Ghana	22,900	3	13.1
South Asia	India, Sri Lanka	220,169	18	8.2
South America	Ecuador, Peru, Bolivia	43,928	3	6.8
Southeast Asia	Philippines	54,231	3	5.5
Southeast Asia	Malaysia	43,698	2	4.6
Central America	Greater Antilles Islands^b^	429,614	9	2.1
Asia	Asia, Southeast Asia, Taiwan, Singapore	No data available	7	–
Australia and Pacific Islands	Australia, Pacific Islands	No data available	6	–
Africa	Africa, Cape Verde Islands, Congo	No data available	3	–
Central America	Mexico	No data available	1	–
			Total: 233^c^	

^a^Lesser Antilles: Antigua and Barbuda, Barbados, Dominica, Grenada, Guadeloupe, Martinique, St. Lucia, St. Vincent, and Grenadines. ^b^Greater Antilles: Bahamas, Cayman Islands, Dominican Republic, Haiti, Jamaica, Cuba. ^c^A total of 233 destinations were mentioned by 225 patients (6 mentioned 2 destinations, l mentioned 3); 5 patients did not provide information about predisease travel, and l case arose in Germany.

**Figure 2:**
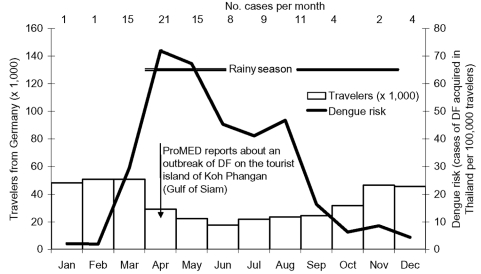
Risk for dengue fever (DF) among travelers to Thailand, 2002. Number within column represents cases per month.

**Figure 3:**
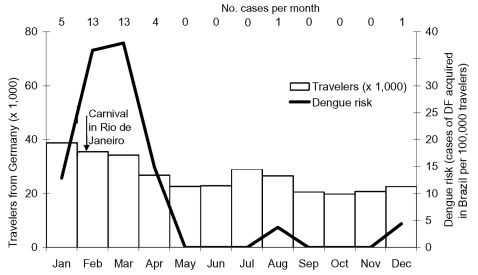
Risk for dengue fever (DF) among German travelers to Brazil, 2002 (Tourism data from 2001). Number within column represents cases per month.

The incidence of dengue fever among German travelers to Thailand ranged from 2/100,000 in January and February to >70/100,000 in April (Figure 2). While travel peaks during the winter months, dengue incidence was strongly elevated during the mid-year rainy season and the month preceding it. Numbers of travelers to Brazil were slightly higher from January to March compared to the rest of the year (Figure 3). The incidence of dengue fever showed a distinct peak in February and March, reaching 39/100,000 travelers in March, and was very low from May to December.

## Conclusions

Some of the steady increase in case reports in early 2001 likely reflects knowledge and acceptance of the recently improved surveillance system among physicians and laboratories who diagnose dengue. The further steep rise in German case reports, particularly during late 2001 and the first half of 2002, corresponds to a surge of local dengue reporting from many dengue–endemic areas and likely reflects a true increase in imported cases. By number of reports of travel-associated infectious diseases, dengue fever is second only to malaria (≈1,000 cases per year, with a 15% drop in cases from 2001 to 2002) in Germany. A parallel voluntary sentinel surveillance system for imported tropical infections in Germany recorded 78 dengue cases in 2001 and 125 cases in 2002, respectively (*5*). For this system, sites include infectious disease and tropical medicine centers, as well as offices of general practitioners specialized in travel medicine. By definition, there is much overlap of cases between the Robert Koch Institute’s mandatory reporting surveillance system, and the voluntary sentinel system. The fact that in 2001 more cases were reported to the sentinel system compared to the institute’s surveillance shows that reporting in the latter system was incomplete in its first year. However, completeness of reporting was much improved in 2002.

The spectrum of countries of infection reflects both predominating travel destinations and local trends in dengue fever endemicity, with risk for individual travelers high in both popular tourist destinations, such as Thailand or Brazil, as well as some regions visited by smaller numbers of travelers, including some Caribbean islands. Within countries endemic for the disease, dengue risk varies by place, season, and year. Urban areas can have intense and prolonged local epidemics. Introduction of a dengue virus serotype for which the population lacks immunity can cause particularly high incidence, and climate may have a strong influence on vector populations. Such fluctuations likely influenced German data.

The increase in cases from the fourth quarter of 2001 to the first quarter of 2002 is mainly due to cases imported from Brazil. During the first quarter of 2002, the state of Rio de Janeiro recorded an incidence that was 6.5 times higher than it had been in January through March of 2001. This state alone accounted for almost 50% of the total cases in Brazil during this period (*6*), including an urban epidemic in the city of Rio de Janeiro. Rio draws large numbers of German tourists, especially during the festival of Carnival, which most likely contributed to the high number of cases acquired in Brazil in February and March 2002 (Figure 3). In contrast, the decrease in incidence in neighboring Venezuela (from 338/100,000 in 2001 [*7*] to 153/100,000 in 2002 [*8*]) corresponded to a significant decrease in the percentage of German travelers who acquired the disease there.

The peak in cases imported to Germany in the second and third quarter of 2002 reflects the dengue season in Thailand and other parts of Southeast Asia. In Thailand, the disease is associated with the rainy season, which varies regionally but in most areas starts around April. In mid-April of 2002, an out-of-season outbreak was reported at the island resort of Koh Phangan (*9*), which may explain the high incidence among German travelers in March and April. Our data highlight the contribution of Southeast Asia as an area where German travelers acquire dengue fever. These findings agree with those from a Swedish case-control study, which identified travel to the Malay Peninsula as an independent risk factor for imported dengue (*10*) and with reports from a European network of institutions of tropical medicine (*11*). Although nosocomial transmission of the virus in dengue-nonendemic areas is rare, the case detected by our surveillance system clearly shows the potential of bloodborne virus transmission and the need to follow strict hygiene precautions when treating dengue patients.

The new infectious disease surveillance system in Germany, based on clinical case definitions and laboratory confirmation, is one of the few national surveillance systems in industrialized countries to include dengue fever with a specific case definition. As asymptomatic and mild infections are known to occur, some proportion of infections will escape diagnosis (*12*,*13*). Dengue is an important differential diagnosis of fever in travelers to endemic areas (*12*,*14*). In a German study, travelers who had fever after returning from dengue-endemic areas had dengue antibody seroprevalence rates of 7% (*15*). As long distance travel expands, increasing numbers of travelers are potentially exposed to dengue viruses and more often exposed sequentially to multiple serotypes of dengue virus, increasing their potential risk for dengue hemorrhagic fever or dengue shock syndrome. Additional serologic studies in representative samples of symptomatic and asymptomatic travelers are needed to investigate the risk in defined areas.

In its second year, Germany’s dengue surveillance demonstrated a rough parallel in the rate of returning travelers with dengue fever and its incidence in the places they had visited. One strength of this study was its analysis of trends on the basis of incidence specific for country of destination. Systematically collected and analyzed surveillance data on imported infections help formulate region-specific travel advice in addition to information on avoiding the vectors of dengue fever.
